# Pretreatment intravoxel incoherent motion histogram metrics and clinical characteristics for prediction of perineural invasion status and survival in patients with rectal cancer

**DOI:** 10.1186/s13244-025-02075-6

**Published:** 2025-09-19

**Authors:** Ao Yang, Hao Xu, Xin Zhang, Ping Zheng, Li Bo Lin, Jie Ke Liu, Peng Zhou, Xiao Li Chen

**Affiliations:** 1 Department of Radiology, Sichuan Clinical Research Center for Cancer, Sichuan Cancer Hospital & Institute, Sichuan Cancer Center, University of Electronic Science and Technology of China, Chengdu, China; 2 GE Healthcare, Shanghai, China; 3 Department of Pathology, Sichuan Clinical Research Center for Cancer, Sichuan Cancer Hospital & Institute, Sichuan Cancer Center, University of Electronic Science and Technology of China, Chengdu, China

**Keywords:** Rectal cancer, Perineural invasion, Intravoxel incoherent motion, Survival analysis

## Abstract

**Objectives:**

To explore intravoxel incoherent motion (IVIM) for evaluation of the perineural invasion (PNI) status and survival in patients with rectal cancer.

**Materials and methods:**

The true diffusion coefficient (D), pseudo-diffusion coefficient (D*), and microvascular volume fraction (*f*) were recorded together with histogram metrics. Differences in IVIM histogram metrics between the PNI-positive group and the PNI-negative group were analyzed. Univariable and multivariable logistic regression analysis were used for model construction. The area under the receiver operating characteristic curve (AUC) was used to assess the diagnostic performance of the models. Histopathology was used as the PNI endpoint. Kaplan–Meier curve analysis was employed to estimate the disease-free survival (DFS) and overall survival (OS) of patients.

**Results:**

A total of 175 patients were retrospectively enrolled in this study. Multivariable logistic regression analysis showed that higher D_median (odds ratio (OR) = 2.036, *p* = 0.003) and D_min (OR = 1.479, *p* = 0.002) and lower *f*_SD (OR = 0.697, *p* < 0.001) and *f*_kurtosis (OR = 0.485, *p* < 0.001) were independently associated with PNI-positive. The combined model showed the best performance in predicting the PNI status with AUCs, sensitivity, specificity, and accuracy of 0.885, 81.67%, 82.61%, and 82.29%, respectively. Kaplan–Meier curves analysis revealed that the patients with higher scores (> −1.12) of the combined model showed relatively lower 2-year DFS (81.6% vs 93.2%, *p* = 0.014) compared to the patients with lower scores (≤ −1.12).

**Conclusion:**

IVIM histogram metrics could predict the PNI status and serve as a preoperative risk stratification tool.

**Critical relevance statement:**

The combination of IVIM histogram metrics and clinical characteristics could discriminate the PNI status and serve as a surrogate for PNI.

**Key Points:**

- The PNI is an important prognostic factor in rectal cancer.
- The IVIM histogram metrics were associated with the PNI status.
- The combination of IVIM and clinical factors could serve as a surrogate for PNI.

**Graphical Abstract:**

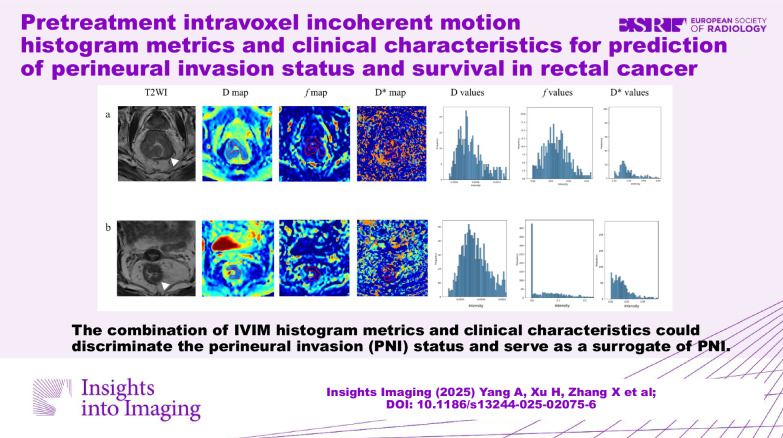

## Introduction

Colorectal cancer (CRC) ranks second in incidence and fourth in mortality, according to the National Cancer Centre of China [1]. Rectal cancers represent over one-third of all CRC cases and are frequently diagnosed at a locally advanced stage, which is defined as a tumor of cT3–4 and/or cN1–2, with no metastatic sites (M0) [2, 3]. Neoadjuvant chemoradiotherapy (nCRT) followed by total mesorectal excision (TME) is a standard treatment for locally advanced rectal cancer [3]. Despite the locoregional recurrence rate having dropped by this treatment, distant metachronous metastases still occur in over 20–30% of cases [4, 5]. Different biological characteristics of CRC may lead to different treatment responses and prognoses [6].

Perineural invasion (PNI) was first described in head and neck cancers, and since then has become an important prognostic marker in different types of cancer, including prostate, pancreas, biliary tract, stomach, and also CRC [7]. PNI is defined as a neoplastic invasion of nervous structures with spread along nerve sheaths [8]. This process may correlate with tumor progression and poor prognosis. A growing body of evidence has shown that the presence of PNI correlates with direct extension, lymphatic metastasis, and hematogenous metastasis [9–12]. Previous studies also demonstrated that PNI status could be an indicator for the selection of patients for nCRT and postoperative adjuvant chemotherapy [13–15]. Therefore, the status of PNI is an important factor for risk stratification, and timely predicting patients with PNI-positive is helpful in tailoring treatment regimes. However, PNI is assessed only histologically at present. Furthermore, there is no correct recommendation to assess PNI on conventional MRI. Thus, an accurate assessment of PNI status prior to surgery is helpful for clinicians to develop individualized treatment plans.

Magnetic resonance imaging (MRI) has been widely used for staging and restaging in rectal cancer [16]. However, conventional MRI usually provides limited information regarding PNI status. Diffusion-weighted imaging (DWI) allows for the characterization of biological tissue by assessing the diffusion motion of the water molecules and quantifying the apparent diffusion coefficient (ADC) [17]. Nevertheless, the ADC shows limited value in evaluating tumor characteristics for its inability to discriminate blood perfusion from the true diffusion of water molecules in the complex tumor microenvironment [18, 19]. Intravoxel incoherent motion (IVIM) has been proposed for this discrimination by multiple *b* values sampling and biexponential curve fit analysis [20]. By using multi-low-*b* values, the IVIM model can segregate effects of perfusion (measurable in the low *b* value range) and true diffusion effects (measurable at high *b* values) [19]. At low *b* values (< 200 s/mm^2^), microvascular volume fraction, *f*, and pseudo-diffusion coefficient, D*, can evaluate the characteristics of perfusion; at high *b* values (≥ 200–1000 s/mm^2^), true diffusion coefficient, D, can reflect water diffusion that is related to tissue cellularity [21–23]. Previous studies have demonstrated the advantages of IVIM for the evaluation of tumor characteristics in rectal cancer, such as nodal metastases, *KRAS* status, extramural vascular invasion, and histological grades [24–27]. Recently, histogram analysis, which is based on pixel distribution, could be used as a quantitative marker of the tumor, such as nodal stage [28, 29]. Therefore, the purpose of the study was to develop models based on IVIM histogram metrics and clinical characteristics to predict the status of PNI in patients with rectal cancer. Furthermore, the disease-free survival (DFS) and overall survival (OS) served as the secondary endpoints, and patients were stratified based on the score of the predictive model to explore the model’s prognostic value.

## Materials and methods

### Patients

The institutional review board of our hospital approved the study (no. SCCHEC-02-2024-049). In this retrospective study, 214 consecutive subjects with a biopsy-proven diagnosis of rectal cancer were enrolled in the study from January 2022 to December 2022. The inclusion criteria of the study were set as follows: (1) MRI examination, including IVIM sequence, was performed; (2) underwent surgical resection within 2 weeks; (3) had complete postoperative pathological data. The exclusion criteria were set as follows: (1) received treatment before surgery (*n* = 19); (2) concurrent distant metastases (*n* = 8); (3) poor image quality (*n* = 3); and (4) incomplete clinical data (*n* = 9).

Clinical characteristics, including age (years), gender, BMI (kg/m^2^), hemoglobin (g/L), albumin (g/L), total protein (g/L), CEA (ng/mL), and CA19.9 (u/mL), were recorded within 1 week prior to the operation.

### MRI protocol

MRI examination was performed with a 3.0-T scanner (Skyra, Siemens, Germany) by using an eight-element phased-array body coil. The imaging protocol (Table S1) comprised sagittal T2WI, oblique coronal T2WI, axial T2WI, and IVIM. The average acquisition time for IVIM is approximately 4 min. No intrarectal gel or spasmolytic agent was used for bowel preparation. All patients emptied their bowels prior to the examination.

### Imaging postprocess and data analysis

First, the acquired IVIM-DWI images with different *b* values were compressed into merged images by MRIcrolGL (v. 1.2, https://www.nitrc.org). Then, the merged images were transferred into MITK (v. 2018.09, https://www.mitk.org) to generate the corresponding parametric maps (D map, *f* map, and D* map), which is free open-source software for the development of interactive medical image processing software and is developed at the German Cancer Research Center (DKFZ). The IVIM parameters were calculated with biexponential curve fit analysis fitting 8 *b* values (*b* = 0 s/mm^2^, 50 s/mm^2^, 80 s/mm^2^, 100 s/mm^2^, 200 s/mm^2^, 500 s/mm^2^, 800 s/mm^2^, 1000 s/mm^2^) according to the following equation described by Le Bihan et al [20]:

$${{{\rm{S}}}}_{{{\rm{b}}}}/{{{\rm{S}}}}_0=(1-f)\cdot \exp (-{{{\rm{b}}}}\cdot {{{\rm{D}}}})+f\cdot \exp ({-{{{\rm{b}}}}\cdot {{{\rm{D}}}}}^{* }),$$

where S_b_ is the signal intensities in the pixel with diffusion gradient b, and S_0_ is the signal intensities in the pixel without diffusion gradient. D is caused by Brownian movement, and *f* and D* are due to microcirculation perfusion.

Two radiologists (Chen X. L. and Yang A., with 15 years and 5 years of experience in pelvic imaging, respectively) who were blinded to the clinical and pathological results independently delineated the whole-tumor volume by manually tracing the outer edge of the lesion on the merged images using ITK-SNAP (v. 3.6.0, http://www.itksnap.org). The intermediate signal-intense lesion area on the T2WI image was selected as the anatomic structure reference. Whole-tumor volume was automatically calculated by summing each of the cross-sectional volumes, which required that the blood vessels, tumor necrosis, and intestinal gas be avoided. Then, the whole-tumor volumes were copied and pasted onto the parametric maps. The IVIM histogram metrics were automatically calculated using PyRadiomics (v. 1.3.0, http://github.com/Radiomics/pyradiomics) based on Python (v. 3.6.3; http://www.python.org) including mean, median, standard deviation (SD), minimum (min), maximum (max), 5th percentile (5th), 10th percentile (10th), 25th percentile (25th), 75th percentile (75th), 90th percentile (90th), 95th percentile (95th), skewness, and kurtosis. The reproducibility of histogram metrics between two observers (Chen X. L. and Yang A.) was evaluated with the intraclass correlation coefficient (ICC) based on the first 20 patients’ data. The data obtained by the first observer were used for further statistical analysis.

### Pathological evaluation

All surgically resected specimens were routinely fixed, and paraffin-embedded sections were stained with hematoxylin-eosin (H&E). The resected specimens, 1 week postoperatively, were evaluated by an experienced gastrointestinal pathologist according to the TNM staging system of the 8th edition of the American Joint Committee on Cancer [30]. PNI was defined as positive, if (1) at least 33% of the nerve circumference was surrounded by cancer cells (without invasion of nerve sheath); or (2) cancer cells were within any layer of the nerve sheath [31, 32].

### Follow-up

Postoperative surveillance was performed every 4 months in the first year and every 8 months in the next year. Evaluations included CEA, CT, or MRI scanning with contrast of the abdominopelvic cavity and CT of the head and chest. Local recurrence was defined as a recurrence in the pelvis, and distant metastasis was defined as a recurrence outside the pelvis.

### Statistical analysis

Statistical analyses were conducted by R software (version 4.0.0, https://www.rproject.org/) and MedCalc (version 18.2.1, https://www.medclac.org). The ICC was calculated to evaluate inter-observer variability for each histogram parameter (0–0.20, poor correlation; 0.21–0.40, fair correlation; 0.41–0.60, moderate correlation; 0.61–0.80, good correlation; and 0.80–1.00, excellent correlation). Continuous variables that followed normal distribution were expressed as mean ± SD, otherwise, their expressed as median and interquartile range. Differences between the groups were analyzed using the independent-sample *t*-test and Mann–Whitney *U*-test according to the Shapiro–Wilk test results. Categorical variables were expressed as counts and compared using the ${{{\rm{\chi }}}}$^2^-test or Fisher’s exact test. Univariate logistic regression analysis was implemented to select independent risk factors, including clinical characteristics and IVIM histogram metrics, for differentiating the PNI status. Subsequently, multivariable binary logistic regression analysis with backward stepwise selection was applied by using the likelihood ratio test as the stopping rule to select variables and construct models. The Hosmer–Lemeshow test was used to assess the goodness-of-fit of the models. Receiver-operating characteristic (ROC) curve analysis and the area under the ROC curve (AUC) were compared using the DeLong test [33]. The optimal cut-off value was determined according to the Youden Index (sensitivity + specificity − 1). The sensitivity, specificity, and accuracy were also calculated. The 2-year DFS and OS were analyzed using Kaplan–Meier curves and compared by the log-rank test. Cox proportional hazard regression was used to calculate the hazard ratio (HR). A two-tailed *p*-value less than 0.05 indicated statistical significance.

## Results

### Patients characteristics

**Fig. 1:**
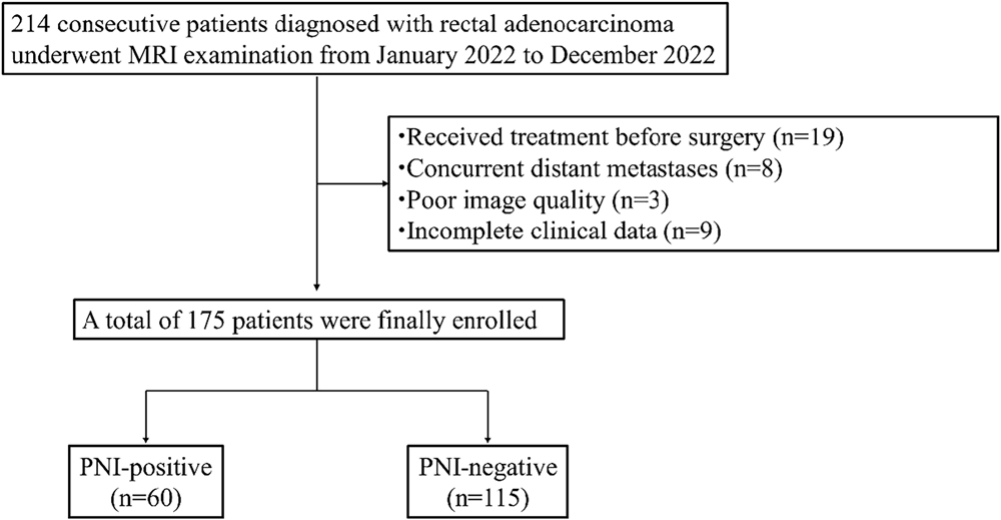
Flowchart of patient inclusion and exclusion criteria

**Fig. 2:**
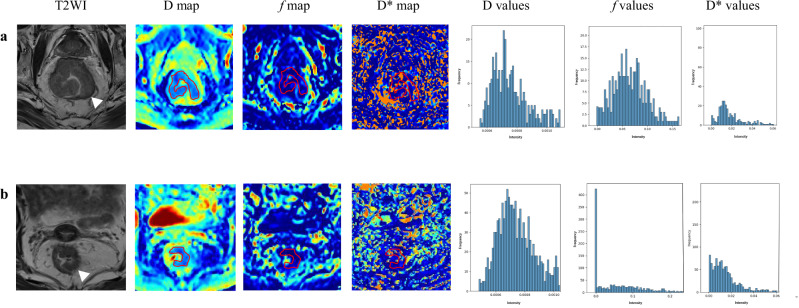
**a** An 80-year-old man diagnosed with rectal cancer confirmed as PNI-positive. **b** A 79-year-old woman diagnosed with rectal cancer confirmed as PNI-negative. D, diffusion coefficient; *f*, microvascular volume fraction; D*, pseudo-diffusion coefficient

After excluding the ineligible patients (*n* = 39), a total of 175 patients were enrolled in this study (Fig. 1). Table 1 shows the baseline characteristics of the study population based on PNI status. The total protein, CA19.9, mrN stage, histological differentiation, pT stage, and pN stage showed significant statistical differences between the two groups (all *p* < 0.05). The representative figures are shown in Fig. 2.

**Table 1:** Baseline characteristics of the patients

Characteristics	PNI-positive (*n *= 60)	PNI-negative (*n* = 115)	*p* value
Age (years)^※^	59.20 ± 11.30	62.19 ± 9.63	0.068
Gender (%)			0.187
Female	27 (45.0%)	40 (34.8%)	
Male	33 (55.0%)	75 (65.2%)	
BMI (kg/m^2^)	23.05 (21.25, 25.27)	23.37 (21.71, 25.11)	0.464
Hemoglobin (g/L) (%)			0.053
≤ 120	23 (38.3%)	28 (24.3%)	
> 120	37 (61.7%)	87 (75.7%)	
Albumin (g/L) (%)			0.101
≤ 37.8	24 (40.0%)	32 (27.8%)	
> 37.8	36 (60.0%)	83 (72.2%)	
Total protein (g/L) (%)			0.004*
≤ 63.4	26 (43.3%)	26 (22.6%)	
> 63.4	34 (56.7%)	89 (77.4%)	
CEA (ng/mL) (%)			0.116
≤ 3.57	28 (46.7%)	68 (59.1%)	
> 3.57	32 (53.3%)	47 (40.9%)	
CA19.9 (u/mL) (%)			0.023*
≤ 14.73	33 (55.0%)	83 (72.2%)	
> 14.73	27 (45.0%)	32 (27.8%)	
mrT stage (%)			0.480
mrT2	7 (11.7%)	16 (13.9%)	
mrT3a	11 (18.3%)	28 (24.3%)	
mrT3b	20 (33.3%)	30 (26.1%)	
mrT3c	7 (11.7%)	18 (15.7%)	
mrT3d	0 (0.0%)	2 (1.7%)	
mrT4	15 (25.0%)	21 (18.3%)	
mrN stage (%)			0.003*
mrN0	15 (25.0%)	55 (47.8%)	
mrN1–2	45 (75.0%)	60 (52.2%)	
Tumor length (cm)	4.90 (3.40, 6.00)	4.40 (3.50, 5.50)	0.360
Thickness (cm)	1.50 (1.30, 1.70)	1.50 (1.30, 1.90)	0.307
Tumor location (%)			0.789
Upper	14 (23.3%)	27 (23.5%)	
Middle	31 (51.7%)	54 (47.0%)	
Lower	15 (25.0%)	34 (29.6%)	
MRF (%)			0.165
Negative	43 (71.7%)	93 (80.9%)	
Positive	17 (28.3%)	22 (19.1%)	
mrEMVI (%)			0.564
Negative	56 (93.3%)	111 (96.5%)	
Positive	4 (6.7%)	4 (3.5%)	
Histological differentiation (%)			< 0.001*
Well-moderate	45 (75.0%)	108 (93.9%)	
poor	15 (25.0%)	7 (6.1%)	
pT stage (%)			< 0.001*
pT1–2	3 (5.0%)	45 (39.1%)	
pT3–4	57 (95.0%)	70 (60.9%)	
pN stage (%)			< 0.001*
pN0	23 (38.3%)	79 (68.7%)	
pN1–3	37 (61.7%)	36 (31.3%)	

^※^ data are means ± SDs

* *p* < 0.05

*BMI* body mass index, *CEA* carcinoembryonic antigen, *CA19.9* carbohydrate antigen 19.9, *MRF* mesorectal fascia, *mrEMVI* extramural vascular invasion in MRI

### Comparison of IVIM histogram metrics between the PNI-positive and PNI-negative groups

**Fig. 3:**
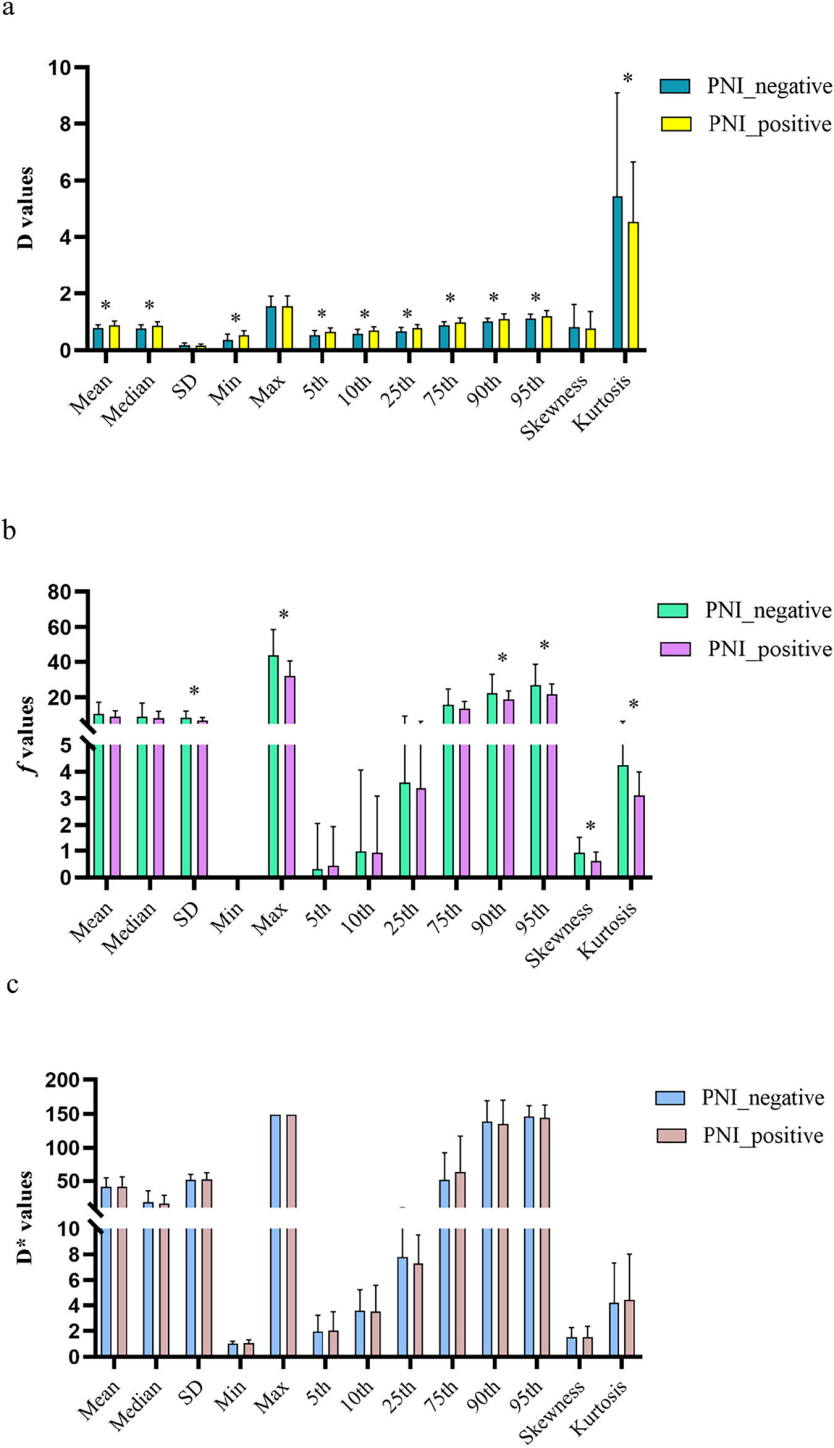
Differences of histogram metrics of D (**a**), *f* (**b**), and D* (**c**) between the positive and negative PNI groups. *Means the differences between the two groups showed a statistical difference

All IVIM histogram metrics showed moderate and good inter-observer agreement except for several extreme metrics (Table S2). The values of mean, median, min, 5th, 10th, 25th, 75th, 90th, and 95th percentiles of the D were significantly higher in the PNI-positive group compared with the PNI-negative group (all *p* < 0.05). The values of kurtosis of the D were lower in the PNI-positive group compared with the PNI-negative group (*p* = 0.028). The values of SD, max, 90th, 95th, skewness, and kurtosis of the *f* were significantly lower in the PNI-positive group compared with the PNI-negative group (all *p* < 0.05). No significant statistical differences were observed between the two groups regarding the histogram metrics of the D*. All the IVIM histogram metrics are shown in Fig. 3 and Table S3.

### Model construction

**Fig. 4:**
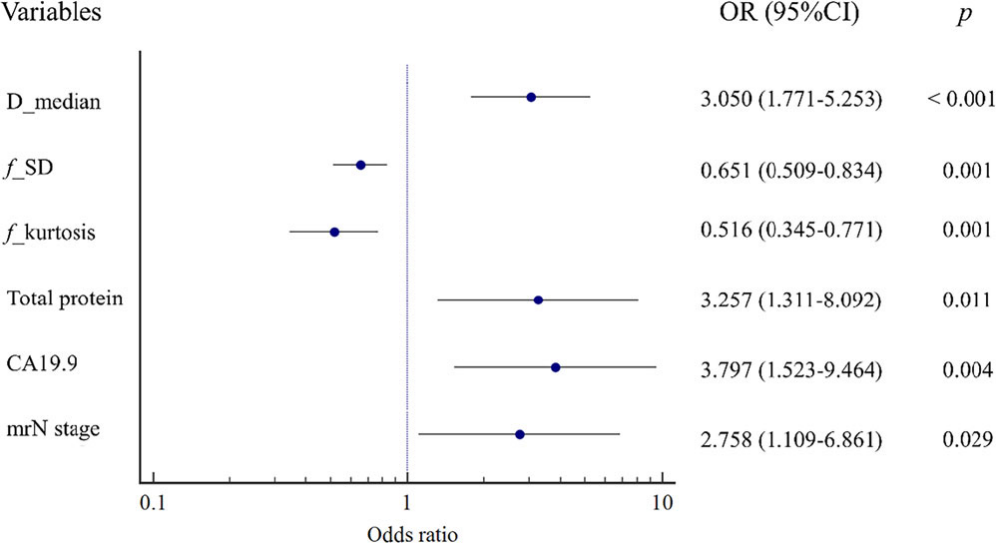
The variables in the combined model for predicting PNI status are shown in a forest plot. The odds ratios (OR) and 95% confidence intervals (CI) for each variable are presented in the figure. The vertical line (OR = 1) indicates the no-effect reference line. Confidence intervals that do not cross this line indicate that the variable is significantly associated with the outcome (*p* < 0.05). Point estimates (circle) size reflects the weight of the variable in the model

Univariable analysis of histogram metrics of D showed that mean, median, min, 5th, 10th, 25th, 75th, 90th, and 95th were associated with the PNI status and thus included in the multivariable analysis. The model based on the D map finally included the median and minimum of the D value. Similarly, univariable analysis of histogram metrics of *f* showed that SD, max, 90th, 95th, skewness, and kurtosis were associated with the PNI status and thus included in the multivariable analysis. The model based on the *f* map finally included the SD and kurtosis of the *f* value (Table 2). To construct the model-integrated D map and *f* map (IVIM model), the *f*_SD, *f*_kurtosis, D_median, and D_min were included in the multivariable logistic regression analysis, and the D_min was finally excluded from the IVIM model. According to the univariable and multivariable analysis, the Clinical model finally included total protein, CA19.9, and mrN stage (Table 3). To construct the combined model, variables of the Clinical model and the IVIM model were entered into the multivariable analysis, and all variables showed significant statistical differences (total protein, CA19.9, mrN stage, *f*_SD, *f*_kurtosis, and D_median). The details of the combined model are shown as a forest plot in Fig. 4. Potential collinearity among variables was tested using the variance inflation factor (VIF), with a VIF ≥ 5, indicating the presence of collinearity. Considering that all VIFs were less than 5, no collinearity was determined among variables (Table S4).

**Table 2:** Univariate and multivariate logistic regression analysis for IVIM histogram metrics

Variable	Univariable analysis		Multivariable analysis	
	OR	*p* value	OR	*p* value
D (×10^−3^ mm^2^/s)				
Mean	2.858 (1.811, 4.510)	< 0.001*	-	-
Median	2.595 (1.696, 3.970)	< 0.001*	2.036 (1.272, 3.259)	0.003*
Min	1.755 (1.383, 2.227)	< 0.001*	1.479 (1.152, 1.900)	0.002*
5th percentile	2.192 (1.522, 3.159)	< 0.001*	-	-
10th percentile	2.542 (1.670, 3.870)	< 0.001*	-	-
25th percentile	2.921 (1.835, 4.650)	< 0.001*	-	-
75th percentile	2.041 (1.451, 2.871)	< 0.001*	-	-
90th percentile	1.610 (1.231, 2.106)	0.001*	-	-
95th percentile	1.282 (1.058, 1.553)	0.011*	-	-
Kurtosis	0.874 (0.753, 1.015)	0.077	-	-
*f* (%)				
SD	0.740 (0.622, 0.881)	0.001*	0.697 (0.576, 0.842)	< 0.001*
Max	0.913 (0.879, 0.948)	< 0.001*	-	-
90th percentile	0.930 (0.879, 0.983)	0.011*	-	-
95th percentile	0.926 (0.880, 0.974)	0.003*	-	-
Skewness	0.272 (0.130, 0.571)	0.001*	-	-
Kurtosis	0.541 (0.392, 0.747)	< 0.001*	0.485 (0.344, 0.685)	< 0.001*

* *p* < 0.05

**Table 3:** Univariate and multivariate logistic regression analysis for clinical characteristics

Variable	Univariable analysis		Multivariable analysis	
	OR	*p* value	OR	*p* value
Total protein	2.618 (1.337, 5.126)	0.005*	2.663 (1.312, 5.402)	0.007*
CA19.9	2.122 (1.106, 4.073)	0.024*	2.349 (1.175, 4.698)	0.016*
mrN stage	2.750 (1.380, 5.480)	0.004*	2.651 (1.299, 5.412)	0.007*

* *p* < 0.05

### Diagnostic performance evaluation

**Fig. 5:**
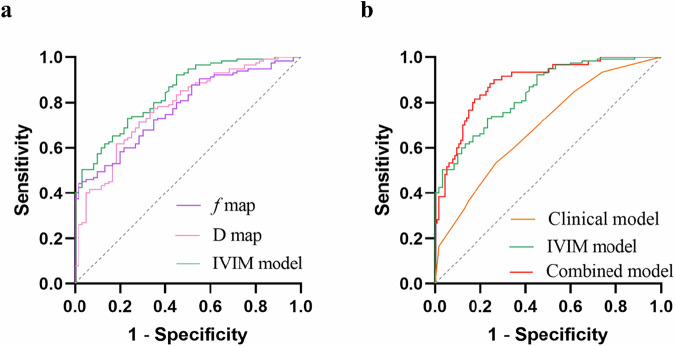
ROC curves of IVIM histogram metrics (**a**) and different models (**b**) for evaluating PNI status

The AUCs of the D map, *f* map, and the Clinical model for discriminating the PNI status are 0.779, 0.774, and 0.691, respectively. The IVIM model showed superior diagnostic performance in identifying the PNI status compared with single map models (D map and *f* map) with AUCs, sensitivity, specificity, and accuracy of 0.841, 76.67%, 73.04%, and 74.29%, respectively. Furthermore, the combined model integrating IVIM histogram metrics and clinical characteristics showed the best diagnostic performance with AUCs, sensitivity, specificity, and accuracy of 0.885, 81.67%, 82.61%, and 82.29%, respectively. The details of the diagnostic performance of the models are shown in Table 4. The ROC curves are shown in Fig. 5.

**Table 4:** Diagnostic performance of models based on IVIM metrics and clinical characteristics for discriminating the PNI status

	AUC	95% CI	Sensitivity (%)	Specificity (%)	Cutoff value	Accuracy (%)
Clinical model	0.691	0.617–0.759	53.33	73.04	−0.92	66.29
D map	0.779	0.710–0.838	81.67	61.74	−0.88	68.57
*f* map	0.774	0.705–0.834	98.33	44.35	−1.52	70.06
IVIM model	0.841	0.779–0.892	76.67	73.04	−0.62	74.29
Combined model	0.885	0.828–0.928	81.67	82.61	−0.47	82.29

### Survival outcomes and subgroup analysis

**Fig. 6:**
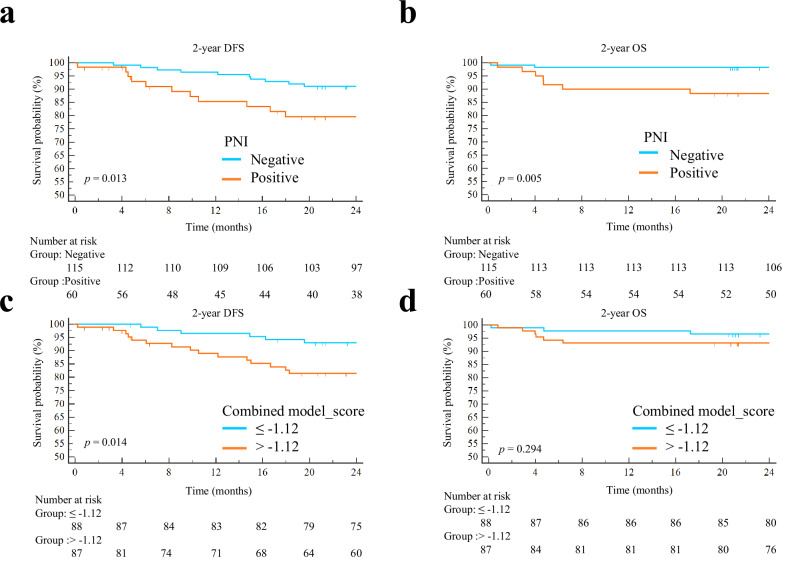
Kaplan–Meier curves for DFS and OS associated with PNI status and the score of the combined model. The patients with PNI-positive showed significantly lower 2-year DFS (**a**) and OS (**b**) compared with the patients with PNI-negative. Furthermore, the score of the combined model was converted into low- and high-risk categories using the median (−1.12). The patients with a higher score (> −1.12) of the combined model showed significantly lower 2-year DFS (**c**) compared with the patients with a lower score (≤ −1.12), while showing no statistical difference in the 2-year OS (**d**)

Median follow-up was 24 months (range, 0.2–24 months). The 2-year OS (88.3% vs 98.3%, *p* = 0.005) and DFS (80.0% vs 91.3%, *p* = 0.013) were significantly lower in the PNI-positive group compared with the PNI-negative group (Fig. 6). The score of the combined model was converted into low- and high-risk categories using the median (−1.12). Kaplan–Meier survival curves and log-rank test revealed that the patients with higher scores (> −1.12) of the combined model showed relatively lower 2-year DFS (81.6% vs 93.2%, *p* = 0.014) compared to the patients with lower scores (≤ −1.12), but the 2-yeas OS between the two groups showed no statistical difference (Fig. 6). Further Cox proportional hazard analysis showed that MRF status (HR 2.681, 95% confidence interval [CI] 1.141–6.300, *p* = 0.024) and the score of the combined model (HR 2.882, 95% CI 1.125–7.383, *p* = 0.027) were independent risk factors for poor 2-year DFS. The combined model_score failed to show significant prognostic power (HR = 2.067, 95% CI = 0.517-8.265, *p* = 0.305) regarding the 2-year OS (Table 5). As for the subgroup analysis, patients with PNI-positive had a lower 2-year DFS than the patients with PNI-negative in the pN0 stage (87.0% vs 97.5%, *p* = 0.0294). Patients with PNI-positive had a lower 2-year OS than the patients with PNI-negative in pT1/2 stage (66.7% vs 97.8%, *p* = 0.0111), pT3/4 stage (89.5% vs 98.6%, *p* = 0.0262), and pN0 stage (87.0% vs 98.7%, *p* = 0.0109) (Fig S1).

**Table 5:** Radiological predictors associated with OS and DFS

Factors	Univariate		Multivariate	
	HR (95% CI)	*p*	HR (95% CI)	*p*
OS				
EMVI	0.046 (0.00, 4679.64)	0.661		
MRF	1.015 (0.211, 4.887)	0.985		
Tumor length	0.794 (0.516, 1.222)	0.295		
Tumor location	0.580 (0.227, 1.482)	0.255		
mrT stage	1.151 (0.784, 1.689)	0.473		
mN stage	1.353 (0.338, 5.411)	0.669		
Combined model	2.067 (0.517, 8.265)	0.305		
DFS				
EMVI	1.086 (0.146, 8.092)	0.936		
MRF	2.876 (1.223, 6.760)	0.015*	2.681 (1.141, 6.300)	0.024*
Tumor length	1.194 (1.025, 1.393)	0.023*	/	/
Tumor location	0.701 (0.259, 1.902)	0.486		
mrT stage	1.315 (1.027, 1.685)	0.030*	/	/
mN stage	3.474 (1.170, 10.311)	0.025*	/	/
Combined model	3.045 (1.191, 7.783)	0.020*	2.882 (1.125, 7.383)	0.027*

## Discussion

In this study, we constructed models based on IVIM histogram metrics and clinical characteristics to predict the PNI status in patients with rectal cancer. The IVIM model showed superior diagnostic performance compared with the single histogram metric model. Furthermore, the combined model integrating IVIM histogram metrics and clinical characteristics showed improved diagnostic performance compared with the IVIM model, indicating the potential advantages of IVIM histogram analysis for the evaluation of PNI status.

Recent studies have developed radiomics models for predicting PNI status in patients with rectal cancer and shed light on the prospect of utilizing radiomics [34–37]. However, the sample size was relatively small and the external validation was ignored, which limited the generalization of the models. Besides, the application of exogenous gadolinium-containing contrast agents may be associated with allergy and nephrogenic systemic fibrosis [38]. Thus, noninvasive and explainable biomarkers are necessary for identifying the PNI status. Wan et al [39] revealed that histogram parameters in zoomed EPI DWI can help predict the PNI status in patients with rectal cancer, which demonstrated the advantages of DWI and histogram analysis in rectal cancer. Compared with conventional DWI, IVIM could separate the blood perfusion from the true diffusion of molecules, which may provide more details about the tumor microenvironment [20].

In the present study, we explored the association between the PNI status and IVIM histogram metrics. Our data demonstrated that the values of mean, median, min, 5th, 10th, 25th, 75th, 90th, and 95th percentiles of the D were significantly higher in the PNI-positive group compared with the PNI-negative group. D is the true diffusion coefficient caused by Brownian movement, which may be correlated to the cellular density, with higher D values indicating lower cellular density. The PNI-positive tumor may have more necrotic or cystic components compared with the PNI-negative tumor because of the unbalanced growth, which may reduce the association between diffusion motion and cellular density, resulting in relatively higher D values [40]. Furthermore, we also found that the kurtosis of the D values was lower in the PNI-positive group compared with the PNI-negative group. Kurtosis reflects the peakedness of the distribution and is a measure of the shape of the probability distribution [41]. In biological tissues, a sharp distribution curve may occur when water motion complies with a Gaussian distribution, suggesting a relatively homogeneous environment [42]. Therefore, the PNI-positive tumor with poor differentiation characteristics may have more heterogeneous components, resulting in a relatively lower kurtosis of the D values compared with the PNI-negative tumor.

As previous studies described [43, 44], the perfusion factor *f* correlated well with vessel area, vessel count, and vessel density, which reflect the level of blood perfusion in the tumor tissue. The correlation between IVIM perfusion-related parameters and dynamic contrast-enhanced MRI quantitative parameters has also been confirmed [45, 46]. The present study demonstrated that the values of SD, max, 90th, 95th, skewness, and kurtosis of the *f* were significantly lower in the PNI-positive group compared with the PNI-negative group. It could be explained by the fact that the tumor with PNI-negative may have a more mature vessel structure compared with the PNI-positive tumor, which contains unstable blood perfusion resulting in a more complex component. However, this result should be interpreted with caution because the *f* value tends to be affected by T2 contributions of both perfusion and pure molecular diffusion compartments [47]. Similar to *f*, perfusion-related parameter D* also reflects the perfusion component in the tumor tissue. Regrettably, the difference of D* between the PNI-positive group and the PNI-negative group showed no statistical significance. It can be explained as follows: First, we used free-breathing DWI, which may introduce blurring. Secondly, the measurement of D* depends upon a number of factors, such as the number of *b* values and signal-to-noise ratio [48]. Thirdly, previous studies also demonstrated that D* appeared to be the least reproducible parameter among IVIM metrics, with the coefficient of variation ranging from 24.8% to 193.8% [49, 50].

A recent study [51] established models based on IVIM histogram analysis and demonstrated that the combined-parameter model shows better performance for diagnosing PNI status compared with the single-parameter model. Furthermore, they integrated IVIM histogram metrics and clinical characteristics to generate the combined model, but unfortunately, the incorporation of the clinical characteristics made little impact. In the present study, we also demonstrated that the IVIM model showed better performance in predicting PNI status compared with single map models. Moreover, we combined the IVIM model and the Clinical model to establish the combined model, which showed superior performance in predicting PNI status compared with the IVIM model and the Clinical model. Additionally, the combined model showed a relatively balanced sensitivity, specificity, and accuracy compared with other models. Thus, our findings suggest that integrating clinical risk factors and IVIM metrics could help to discriminate the PNI status in patients with rectal cancer.

The present study demonstrated that the 2-year OS and DFS were significantly lower in the PNI-positive group compared with the PNI-negative group, which demonstrated that PNI positivity was a significant prognostic factor in rectal cancer. Further subgroup analysis showed that in the N0 patients, PNI-positive patients showed poor 2-year OS and DFS compared with PNI-negative patients. Consequently, PNI is a significant prognostic factor in early-stage patients with rectal cancer. Previous studies also revealed a similar prognostic significance of PNI-positive [4, 9]. Besides, our results suggested that the combined model could predict PNI status, and the score of the combined model was a risk factor for 2-year DFS in patients with rectal cancer. Thus, the combined model may serve as a surrogate for PNI-positive and for risk stratification prior to surgery.

The COX analysis conducted in this study cohort indicated poor prognostic efficacy of EMVI. The possible reasons for this conflict result may be found as follows: Firstly, the evaluation of EMVI may be greatly affected by radiologists’ experience. EMVI should be reported as present if the normal dark vascular flow void on T2WI is replaced by an intermediate signal intensity similar to that of the primary rectal tumor [52]. However, the differentiation between the tumors in lymphatics and extramural venous remains challenging as they share the same characteristics of a serpiginous extension of the tumor signal and the mesorectum [53]. A previous study reported an ICC ranging from 0.38 to 0.69 and AUCs for diagnosis ranging from 0.66 to 0.83 when assessing the EMVI based on T2WI [54]. Secondly, the sensitivity of conventional MR for the detection of EMVI was relatively lower, ranging from 40% to 72% [55–58]. Furthermore, the added value of DWI and contrast-enhanced imaging in detecting EMVI is conflicting [59]. Thus, the detection of EMVI remains challenging in clinical practice. Finally, the assessment of EMVI was recorded as a binary result of positive or negative in the present study. However, as previous studies pointed out, not only the presence, but also the size and number of EMVIs may be significant clinically [60].

This study has a few limitations. Firstly, this was a retrospective single-center study with a relatively small sample size, which may result in potential selection bias. Prospective and multi-center studies with larger sample sizes are needed to validate the model in the future. Secondly, whole-tumor volume segmentation was performed manually, which could be time-consuming. Thirdly, the time of follow-up was relatively short. Our further study plans to prolong the time of follow-up.

In conclusion, the histogram metrics of IVIM could help to discriminate the PNI status, and the model based on IVIM and clinical risk factors could be used as a preoperative risk stratification tool in patients with rectal cancer.

## Supplementary information

ELECTRONIC SUPPLEMENTARY MATERIAL

## Data Availability

All data are with the corresponding author and can be obtained by mail if necessary.

## Abbreviations

AUC: Area under the ROC curve
CRC: Colorectal cancer
D: Diffusion coefficient
DFS: Disease-free survival
D*: Pseudo-diffusion coefficient
*f*: Microvascular volume fraction
OS: Overall survival
PNI: Perineural invasion
ROC: Receiver-operating characteristic
