# Supplementary material for: Pretreatment intravoxel incoherent motion histogram metrics and clinical characteristics for prediction of perineural invasion status and survival in patients with rectal cancer
Source: Insights Imaging. 2025 Sep 19;16:199. doi: 10.1186/s13244-025-02075-6 (PMC12449288; doi:10.1186/s13244-025-02075-6)
Supplement: Supplementary file 1 — ELECTRONIC SUPPLEMENTARY MATERIAL [file 13244_2025_2075_MOESM1_ESM.pdf]

Pretreatment intravoxel incoherent motion histogram metrics and clinical characteristics for prediction of perineural invasion status and survival in rectal cancer

ELECTRONIC SUPPLEMENTARY MATERIAL

Table S1 The MRI protocol of this study

Parameters	Sagittal T2WI	Oblique coronal T2WI	Axial T2WI	IVIM
Scanning sequence	SE	SE	SE	EP
Slice thickness (mm)	3	3	3	4
Spacing between slices (mm)	3.3	3.3	3.3	4.8
TR (ms)	5990	5830	7200	5100
TE (ms)	101	101	101	63
Number of average	2	2	2	2
Echo train length	25	25	25	41
Field of view (mm)	100	100	100	81.25
Matrix	320 × 310	320 × 320	320 × 320	128 × 94
Flip angle	160	160	160	90
b value (s/mm^2)	···	···	···	0, 50, 80, 100, 200, 500, 800, and 1000

Table S2 Interobserver variability of IVIM histogram metrics

	ICC (95% CI)	*p* value
D		
Mean	0.780 (0.497, 0.909)	$p < 0.001$
Median	0.832 (0.594, 0.932)	$p < 0.001$
SD	0.781 (0.523, 0.907)	$p < 0.001$
Min	0.764 (0.472, 0.902)	$p < 0.001$
Max	0.206 (-0.145, 0.553)	0.129
5th percentile	0.672 (0.349, 0.855)	$p < 0.001$
10th percentile	0.818 (0.596, 0.924)	$p < 0.001$
25th percentile	0.860 (0.651, 0.944)	$p < 0.001$
75th percentile	0.792 (0.469, 0.918)	$p < 0.001$
90th percentile	0.736 (0.437, 0.887)	$p < 0.001$
95th percentile	0.738 (0.457, 0.887)	$p < 0.001$
Skewness	0.682 (0.358, 0.860)	$p < 0.001$
Kurtosis	0.555 (0.154, 0.797)	0.005
f (%)		
Mean	0.875 (0.712, 0.949)	$p < 0.001$
Median	0.850 (0.659, 0.938)	$p < 0.001$
SD	0.899 (0.765, 0.958)	$p < 0.001$
Min	-	-
Max	0.587 (0.187, 0.817)	0.001
5th percentile	0.986 (0.966, 0.995)	$p < 0.001$
10th percentile	0.729 (0.432, 0.883)	$p < 0.001$
25th percentile	0.808 (0.583, 0.919)	$p < 0.001$
75th percentile	0.881 (0.724, 0.951)	$p < 0.001$
90th percentile	0.887 (0.737, 0.954)	$p < 0.001$
95th percentile	0.888 (0.744, 0.954)	$p < 0.001$
Skewness	0.606 (0.225, 0.824)	0.002
Kurtosis	0.937 (0.849, 0.975)	$p < 0.001$
D^* ($\times 10^{-3} mm^2/s$)		
Mean	0.799 (0.558, 0.915)	$p < 0.001$
Median	0.664 (0.326, 0.852)	0.001
SD	0.849 (0.663, 0.937)	$p < 0.001$
Min	-0.044 (-0.496, 0.408)	0.572
Max	-	-
5th percentile	0.686 (0.369, 0.861)	$p < 0.001$
10th percentile	0.755 (0.457, 0.897)	$p < 0.001$
25th percentile	0.606 (0.236, 0.823)	0.002
75th percentile	0.835 (0.636, 0.931)	$p < 0.001$
90th percentile	0.638 (0.282, 0.839)	0.001
95th percentile	0.889 (0.745, 0.954)	$p < 0.001$
Skewness	0.870 (0.703, 0.947)	$p < 0.001$
Kurtosis	0.892 (0.748, 0.956)	$p < 0.001$

Table S3 Comparison of IVIM histogram metrics between the positive and negative PNI groups

	PNI positive (n=60)	PNI negative (n=115)	*p* value
D ($\times 10^{-3}mm^2/s$)			
Mean	0.85 (0.81, 0.91)	0.80 (0.75, 0.85)	< 0.001*
Median	0.84 (0.78, 0.91)	0.78 (0.73, 0.83)	< 0.001*
SD	0.16 (0.13, 0.19)	0.17 (0.14, 0.22)	0.160
Min	0.53 (0.46, 0.61)	0.41 (0.23, 0.53)	< 0.001*
Max	1.47 (1.31, 1.77)	1.49 (1.28, 1.77)	0.895
5th percentile	0.65 (0.61, 0.71)	0.58 (0.49, 0.65)	< 0.001*
10th percentile	0.70 (0.64, 0.75)	0.62 (0.55, 0.68)	< 0.001*
25th percentile	0.76 (0.71, 0.82)	0.69 (0.64, 0.74)	< 0.001*
75th percentile	0.96 (0.88, 1.03)	0.88 (0.83, 0.95)	< 0.001*
90th percentile	1.07 (0.99, 1.18)	1.01 (0.94, 1.08)	0.001*
95th percentile	1.15 (1.06, 1.26)	1.11 (1.01, 1.21)	0.016*
Skewness※	0.77 ± 0.60	0.82 ± 0.79	0.657
Kurtosis	3.77 (3.17, 5.37)	4.61 (3.50, 6.15)	0.028*
f (%)			
Mean	8.91 (7.18, 10.48)	9.86 (7.86, 11.53)	0.065
Median	8.30 (6.56, 9.92)	8.60 (6.21, 11.04)	0.642
SD	6.48 (5.85, 7.82)	7.88 (6.49, 9.77)	< 0.001*
Min	0.00 (0.00, 0.00)	0.00 (0.00, 0.00)	> 0.99
Max	28.98 (25.92, 39.66)	41.67 (32.96, 51.51)	< 0.001*
5th percentile	0.00 (0.00, 0.00)	0.00 (0.00, 0.00)	0.354
10th percentile	0.00 (0.00, 0.53)	0.00 (0.00, 0.74)	0.951
25th percentile	2.90 (0.34, 4.92)	2.75 (0.00, 5.10)	0.421
75th percentile	13.20 (11.27, 15.25)	14.06 (11.81, 16.84)	0.091
90th percentile	17.50 (15.27, 19.97)	19.61 (17.14, 24.68)	0.004*
95th percentile	20.87 (18.06, 24.39)	23.52 (20.47, 29.80)	0.001*
Skewness	0.62 (0.38, 0.90)	0.89 (0.59, 1.26)	< 0.001*
Kurtosis	2.95 (2.41, 3.62)	3.63 (2.85, 5.12)	< 0.001*
D^* ($\times 10^{-3}mm^2/s$)			
Mean	38.76 (31.09, 51.18)	38.10 (33.40, 44.95)	0.902
Median	12.92 (11.43, 15.90)	14.41 (11.43, 17.39)	0.306
SD	52.96 (46.41, 59.88)	52.14 (47.59, 56.49)	0.538
Min	1.00 (1.00, 1.00)	1.00 (1.00, 1.00)	0.504
Max	148.51 (148.51, 148.51)	148.51 (148.51, 148.51)	> 0.99
5th percentile	1.00 (1.00, 2.49)	1.00 (1.00, 2.49)	0.823
10th percentile	3.68 (2.15, 5.10)	3.98 (2.49, 4.28)	0.463
25th percentile	6.96 (5.47, 8.45)	6.96 (6.96, 8.45)	0.160
75th percentile	31.55 (23.44, 144.41)	33.78 (25.96, 56.13)	0.970
90th percentile	148.51 (148.51, 148.51)	148.51 (148.51, 148.51)	0.562
95th percentile	148.51 (148.51, 148.51)	148.51 (148.51, 148.51)	0.208
Skewness	1.53 (0.93, 1.98)	1.54 (1.17, 1.86)	0.690
Kurtosis	3.50 (2.04, 5.20)	3.57 (2.59, 4.75)	0.640

※data are means ± standard deviations

Table S4 Collinearity and correlation of the Combined model

	VIF	D_median	*f*_SD	*f*_ kurtosis	Total protein	CA19.9	mrN stage
D_median	1.224	1.000	-0.141* (p = 0.063)	-0.077* (p = 0.310)			
*f*_SD	1.243	/	/	-0.048* (p = 0.529)			
*f*_ kurtosis	1.023	/	/	/			
Total protein	1.014				1.000	-0.041& (p = 0.593)	0.097& (p = 0.201)
CA19.9	1.005				/	/	0.015& (p = 0.845)
mrN stage	1.023				/	/	/

VIF = variance inflation factor, *Spearman's rank correlation coefficient, &Kendall's coefficient

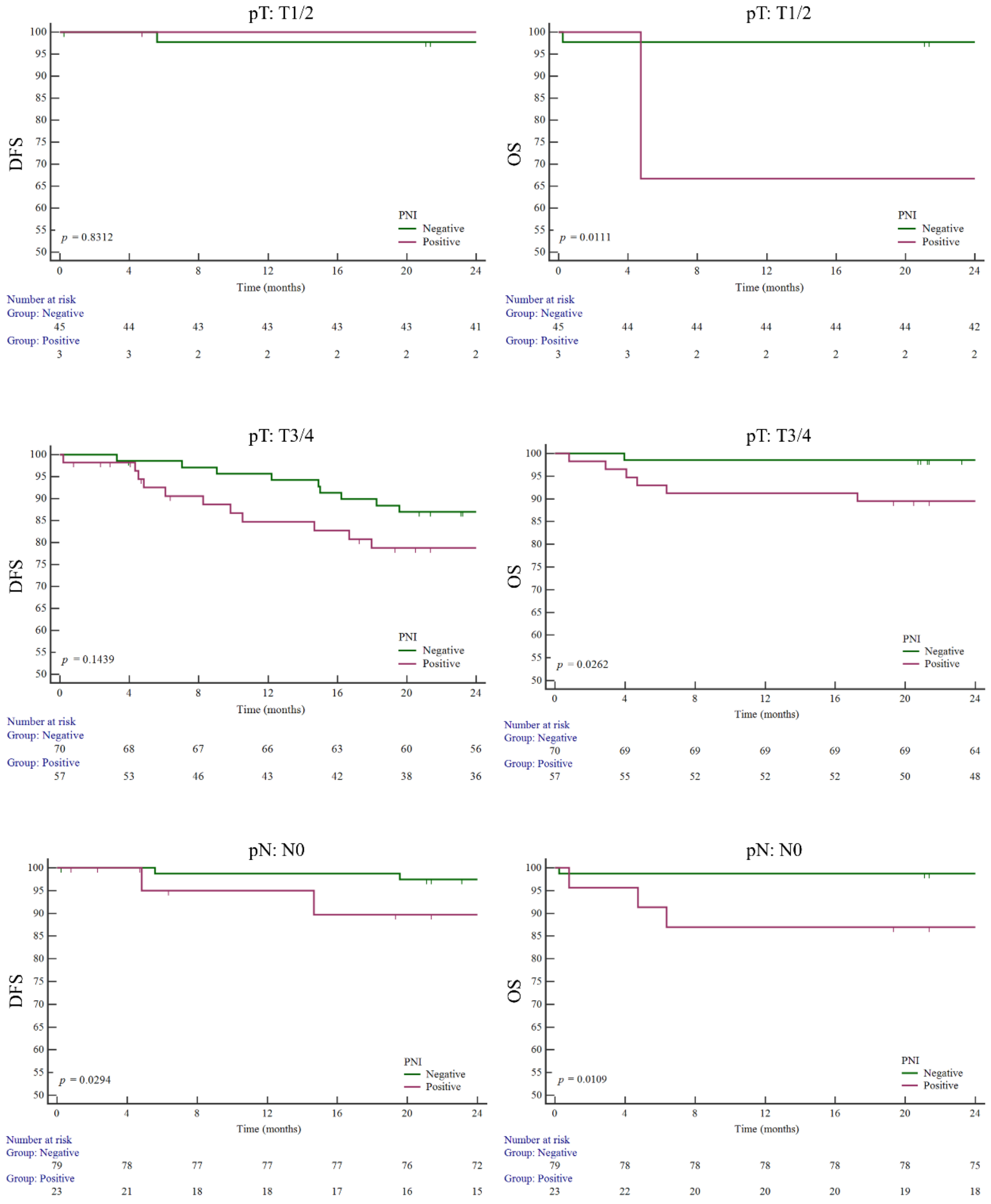
pT: T1/2
DFS
p = 0.8312
PNI
Negative
Positive
Time (months)
Number at risk
Group: Negative
45 44 43 43 43 43 41
Group: Positive
3 3 2 2 2 2 2
pT: T1/2
OS
p = 0.0111
PNI
Negative
Positive
Time (months)
Number at risk
Group: Negative
45 44 44 44 44 44 42
Group: Positive
3 3 2 2 2 2 2
pT: T3/4
DFS
p = 0.1439
PNI
Negative
Positive
Time (months)
Number at risk
Group: Negative
70 68 67 66 63 60 56
Group: Positive
57 53 46 43 42 38 36
pT: T3/4
OS
p = 0.0262
PNI
Negative
Positive
Time (months)
Number at risk
Group: Negative
70 69 69 69 69 69 64
Group: Positive
57 55 52 52 52 50 48
pN: N0
DFS
p = 0.0294
PNI
Negative
Positive
Time (months)
Number at risk
Group: Negative
79 78 77 77 77 76 72
Group: Positive
23 21 18 18 17 16 15
pN: N0
OS
p = 0.0109
PNI
Negative
Positive
Time (months)
Number at risk
Group: Negative
79 78 78 78 78 78 75
Group: Positive
23 22 20 20 20 19 18

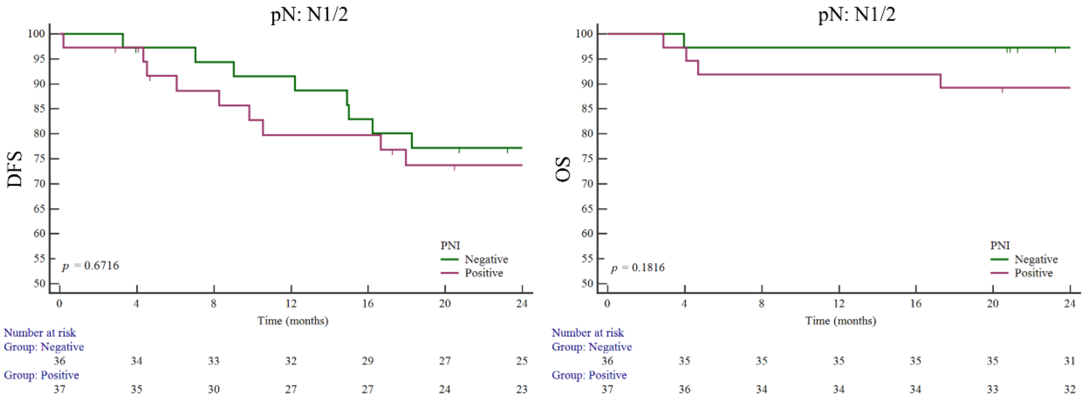

Fig S1 Kaplan-Meier curves of disease free survival (DFS) and overall survival (OS) in various subgroups. pT = pathologic T, pN = pathologic N.
